# Supplementary material for: Short and long-term costs of inbreeding in the lifelong-partnership in a termite
Source: Commun Biol. 2022 Apr 25;5:389. doi: 10.1038/s42003-022-03317-9 (PMC9038770; doi:10.1038/s42003-022-03317-9)
Supplement: Supplementary file 2 — Supplementary Information [file 42003_2022_3317_MOESM2_ESM.pdf]

Short and long-term costs of inbreeding in the lifelong-partnership in a termite

Pierre-André Eyer*, Edward L. Vargo[1]

[1]Department of Entomology, 2143 TAMU, Texas A&M University, College Station, Texas, 77843-2143, USA

Supplementary Methods – DNA extraction and PCR protocols

Termite DNA Extraction:

1. Individual termite workers were placed in a 1.5mL tube.
2. 100 µL of cell lysis solution (10mM Tris, 2% SDS, 0.1M NaCl, 10mM EDTA, 40mM DTT) and 1 µL of proteinase K were added to each tube.
3. Samples were ground in solution using a pestle.
4. Samples were placed in a water bath at 55°C for 3 hours.
5. Samples were removed from the water bath and kept in a freezer at -20°C for 30 minutes.
6. 35 µL of 8M ammonium acetate was then added to thawed samples.
7. Samples were centrifuged for 7 minutes at 10K rpm.
8. The supernatant was pipetted into new tubes containing 100 µL cold isopropanol.
9. Samples were then centrifuged for 5 minutes at 10K rpm.
10. Isopropanol was poured out of tubes, leaving behind pelleted DNA.
11. 400 µL 100% Ethanol was added to each sample.
12. Samples were centrifuged for 5 minutes at 10K rpm.
13. Ethanol was poured out, and samples were placed in a vacuum for 15 minutes to remove any excess alcohol.
14. Dry DNA pellets were resuspended in 100 µL of 1X TE buffer overnight.

Termite 16S PCR:

Reaction Template

**Reagent**	**Per Sample**
DNA template	2.0 µL
Forward Primer	0.2 µL
Reverse Primer	0.2 µL
5x PCR Buffer	5 µL
Taq Polymerase	0.06 µL
Water	18.54 µL

Primer sequences and thermocycler programs

Termite - 16S

LR-J-13007 (5'–TTACGCTGTTATCCCTAA-3')

LR-N-13398 (5'-CGCCTGTTTATCAAAAACAT-3')

94°C for 2 min; 41 cycles of 94°C for 45 sec, 50°C for 45 seconds, 72°C for 1 min; final extension 72°C for 5 min.

Phenol:Chloroform extraction protocol for termite cuticle extraction

1. Pipette 250µL Cell Lysis Buffer (100mM Tric-Cl, 2% CTAB, 1.4M NaCl, 1% PEG 6000, 20mM EDTA) and 3µL Proteinase K into each wash sample. Flick sample tubes to mix.
2. Incubate samples at 37°C overnight.
3. Pipette 3µL of Proteinase K into each sample. Flick sample tubes to mix.
4. Place samples in 65°C water bath for 1 hour.
5. Repeat steps 3-4.
6. Pipette 250µL of phenol:chloroform into each sample. Shake samples under fume hood for 5 minutes.
7. Centrifuge at 12,000 x g for 4 minutes.
8. Pipette supernatant into a new Eppendorf tube.
9. Repeat steps 6-8.
10. Pipette 250µL Chloroform into each sample. Shake samples under fume hood for 5 minutes.
11. Centrifuge at 12,000 x g for 4 minutes.
12. Pipette supernatant into a new Eppendorf tube.
13. Pipette 65µL of Ammonium Acetate and 500µL of ice cold 95% Ethanol into each sample. Flick samples to mix.
14. Place samples in freezer overnight.
15. Centrifuge for 10 minutes at 12,000 x g.
16. Pour off excess ethanol.
17. Pipette 100µL of 70% ethanol into each sample.
18. Repeat steps 15-16.
19. Let samples air dry to remove excess ethanol
20. Pipette 50µL ddH$_2$0 into each sample and incubate at room temperature overnight.
21. Store samples in freezer.

PCR protocols for bacteria and fungi

Reaction Template

Reagent	Per Sample
DNA template	1.0 μL
Forward primer	0.5 μL
Reverse primer	0.5 μL
2x Master mix	5 μL
Water	13.0 μL
Total volume	20.0 μL

Primer sequences and thermocycler programs

Bacteria - 16S v4

515F (5'– GTGCCAGCMGCCGCGGTAA-3')

BAC806R (5'- GGACTACHVGGGTWTCTAAT-3')

94°C for 3 min; 35 cycles of 94°C for 45 sec, 50°C for 60 sec, 72°C for 90 sec; final extension 72°C for 10 min.

Fungi - ITS2 gene region

ITS3 (5'- GCATCGATGAAGAACGCAGC -3')

ITS4 (5'- TCCTCCGCTTATTGATATGC -3')

95°C for 2 min; 40 cycles of 95°C for 30 sec, 55°C for 30 sec, 72°C for 1 min; final extension 72°C for 10 min.

Supplementary Note 1: Potential presence of internal parasites, not counted in the microbial load

Interestingly, alates from colony E had the highest mortality rate in our study, despite not showing the highest level of microbial load. First, this result may reflect the presence of highly virulent strains of fungal or bacterial entomopathogens, despite their modest concentration. Second, movements were frequently observed within the dead bodies of alates from this colony while determining the sex of the dead alates in outbred colonies (Figure 2c; *pers. obs.*). These movements likely originate from internal parasites, such as entomopathogenic nematodes, which are known to occur and to induce high mortality in *R. flavipes*[1], despite not being counted in the microbial load. The presence of fungal or bacterial spores on the surface of the infected partner can be removed by grooming between alates[2-4], as observed in *Z. angusticollis*, where grooming between dealates enables them to control low pathogen exposure of a cuticular fungus[5]. In contrast, the presence of internal parasites is probably harder to overcome by immune behaviors of the partner, even when paired with a resistant partner (*e.g.,* paring AE). This finding also suggests that the selective pressure for the avoidance of a sick partner should not only be based on the detection of the external presence of fungus spores or bacteria, but also on an overall evaluation of partner health, such as changes in behavior or cuticular hydrocarbons[6-8].

References:

1. Nguyen, K.B. and G.C. Smart, *Neosteinernema longicurvicauda n. gen., n. sp. (Rhabditida: Steinernematidae), a parasite of the termite Reticuldermes flavipes (Koller).* Journal of nematology, 1994. **26**(2): p. 162-174.

2. Chouvenc, T., N.-Y. Su, and A. Robert, *Inhibition of Metarhizium anisopliae in the alimentary tract of the eastern subterranean termite Reticulitermes flavipes.* Journal of Invertebrate Pathology, 2009. **101**(2): p. 130-136.

3. Liu, L., et al., *The Influence of Allogrooming Behavior on Individual Innate Immunity in the Subterranean Termite Reticulitermes chinensis (Isoptera: Rhinotermitidae).* Journal of Insect Science, 2019. **19**(1).

4. Yanagawa, A., et al., *Odor aversion and pathogen-removal efficiency in grooming behavior of the termite Coptotermes formosanus.* Plos One, 2012. **7**(10): p. e47412.

5. Rosengaus, R.B., et al., *Mate preference and disease risk in Zootermopsis angusticollis (Isoptera: Termopsidae).* Environmental Entomology, 2011. **40**(6): p. 1554-1565.

6. Beani, L., et al., *Cuticular hydrocarbons as cues of sex and health condition in Polistes dominula wasps.* Insectes Sociaux, 2019. **66**(4): p. 543-553.

7. Cappa, F., et al., *Bee guards detect foreign foragers with cuticular chemical profiles altered by phoretic varroa mites.* Journal of Apicultural Research, 2016. **55**(3): p. 268-277.

8. Trabalon, M., et al., *Modification of morphological characters and cuticular compounds in worker ants Leptothorax nylanderi induced by endoparasites Anomotaenia brevis.* Journal of Insect Physiology, 2000. **46**(2): p. 169-178.

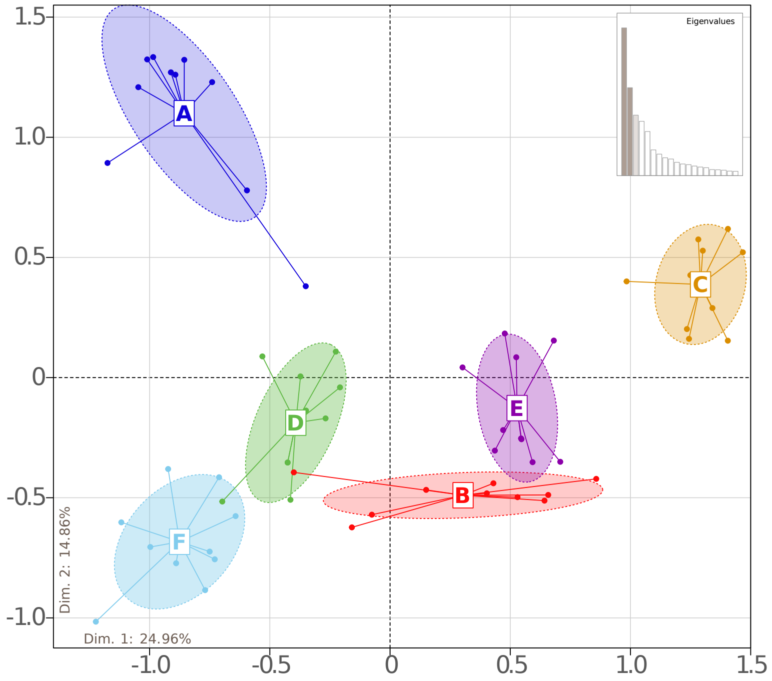

Figure S1

PCA analysis based on microsatellite markers confirming that the nests sampled belong to distinct colonies. Dots represent individuals, each dot is colored according to its nest of origin. Individuals from nests belonging to the same colony are expected to cluster together, while individuals from different colonies should segregate across the axes.

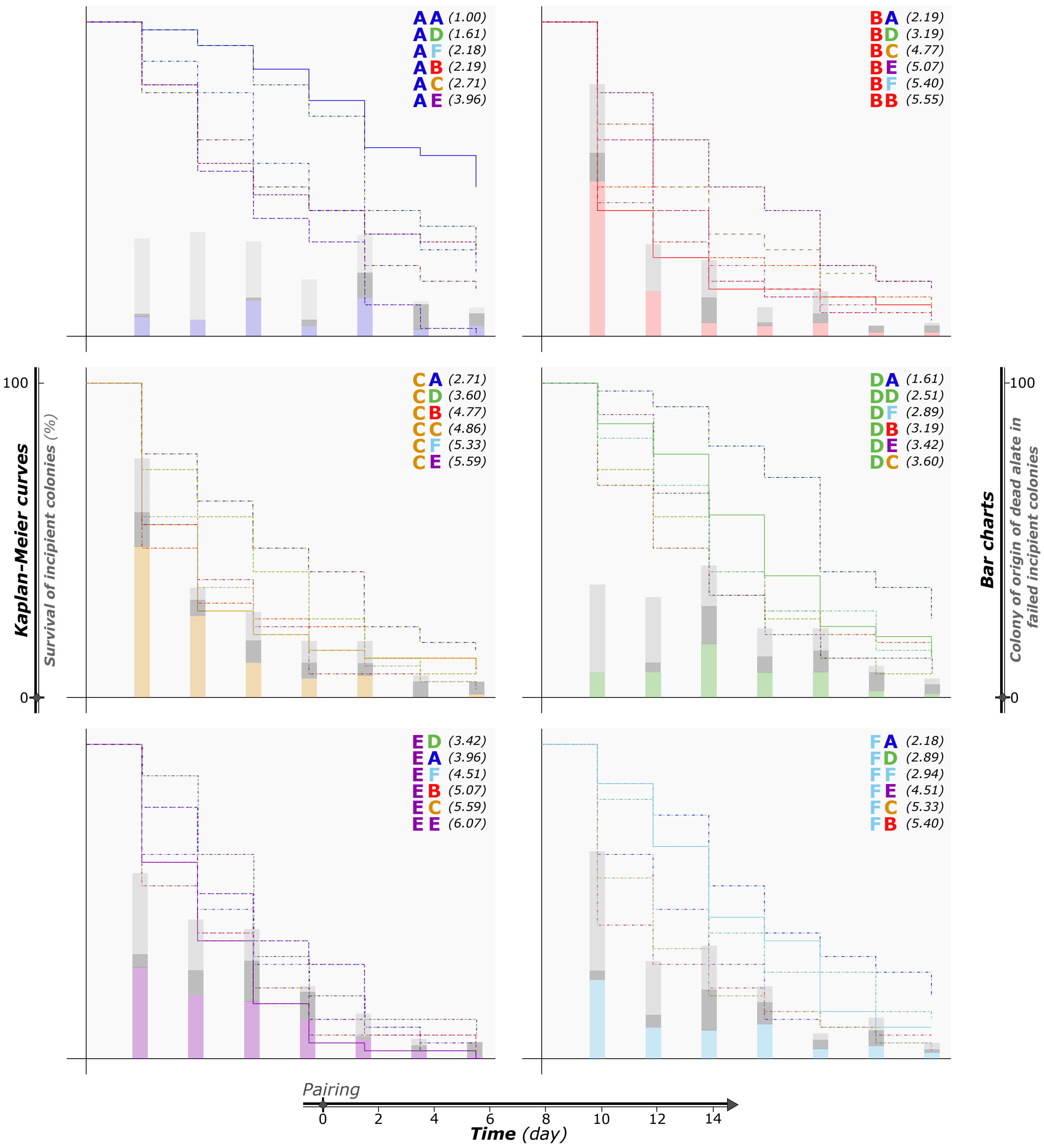

Figure S2: Survival Kaplan-Meier curves for each pairing during the 14 first days of colony establishment (i.e., short-term survival). Incipient colonies were monitored every two days. Hazard ratio for cox-proportional hazard model is reported in front of each pairing. At each census date, bar charts indicate colony of origin of the dead alate(s) in failed incipient colonies. Color bars denote dead alates originating from the studied colony, dark grey bars indicate the death of both alates (i.e., studied colony and partner), and light grey bars represent death of the partner.

Hazard ratio

	A	B	C	D	E	F
A	1.00					
B	2.19	5.55				
C	2.71	4.77	4.86			
D	1.61	3.19	3.60	2.51		
E	3.96	5.07	5.59	3.42	6.07	
F	2.18	-5.40	5.33	2.89	4.51	2.94

Bacterial *Unweighted Unifrac*

	A	B	C	D	E	F
A	0.551					
B	0.675	0.526				
C	0.530	0.701	0.385			
D	0.477	0.598	0.418	0.310		
E	0.561	0.479	0.581	0.440	0.327	
F	0.506	0.712	0.410	0.383	0.569	0.361

Relatedness

	A	B	C	D	E	F
A	0.48 (0.057)					
B	-0.23 (0.019)	0.43 (0.036)				
C	-0.34 (0.014)	0.03 (0.039)	0.75 (0.011)			
D	-0.03 (0.021)	0.05 (0.019)	-0.24 (0.012)	0.52 (0.040)		
E	-0.28 (0.022)	0.02 (0.067)	0.15 (0.040)	-0.01 (0.022)	0.71 (0.035)	
F	-0.12 (0.015)	-0.09 (0.028)	-0.42 (0.015)	0.01 (0.018)	-0.25 (0.063)	0.54 (0.027)

Bacterial *Weighted Unifrac*

	A	B	C	D	E	F
A	0.233					
B	0.174	0.082				
C	0.358	0.285	0.486			
D	0.271	0.282	0.455	0.277		
E	0.243	0.226	0.396	0.284	0.295	
F	0.208	0.202	0.376	0.242	0.240	0.207

Cumulative pathogen load

	A	B	C	D	E	F
A	0.72					
B	15.22	29.72				
C	12.64	27.14	24.56			
D	0.75	15.25	12.67	0.78		
E	4.39	18.89	16.31	4.42	8.06	
F	0.94	15.44	12.86	0.97	4.61	1.16

Fungal *Unweighted Unifrac*

	A	B	C	D	E	F
A	0.653					
B	0.736	0.797				
C	0.642	0.731	0.751			
D	0.524	0.612	0.542	0.453		
E	0.751	0.650	0.717	0.614	0.592	
F	0.679	0.666	0.657	0.529	0.640	0.683

Maximum pathogen load

	A	B	C	D	E	F
A	0.36					
B	14.86	14.86				
C	12.28	14.86	12.28			
D	0.39	14.86	12.28	0.39		
E	4.03	14.86	12.28	4.03	4.03	
F	0.58	14.86	12.28	0.58	4.03	0.58

Fungal *Weighted Unifrac*

	A	B	C	D	E	F
A	0.188					
B	0.268	0.258				
C	0.217	0.215	0.089			
D	0.195	0.248	0.193	0.163		
E	0.284	0.242	0.143	0.257	0.127	
F	0.296	0.270	0.201	0.284	0.197	0.222

Figure S3: Hazard ratio for each pairing in the first 14 days after colony establishment. Additional matrices provide values of every variable tested for each pair of colonies (maximum pathogen load, cumulative pathogen load, relatedness (variance is indicated in brackets), unweighted Unifrac bacterial difference, weighted Unifrac bacterial difference, unweighted Unifrac fungal difference and weighted Unifrac fungal difference. These values are used to test for the correlations presented in Figure 3).

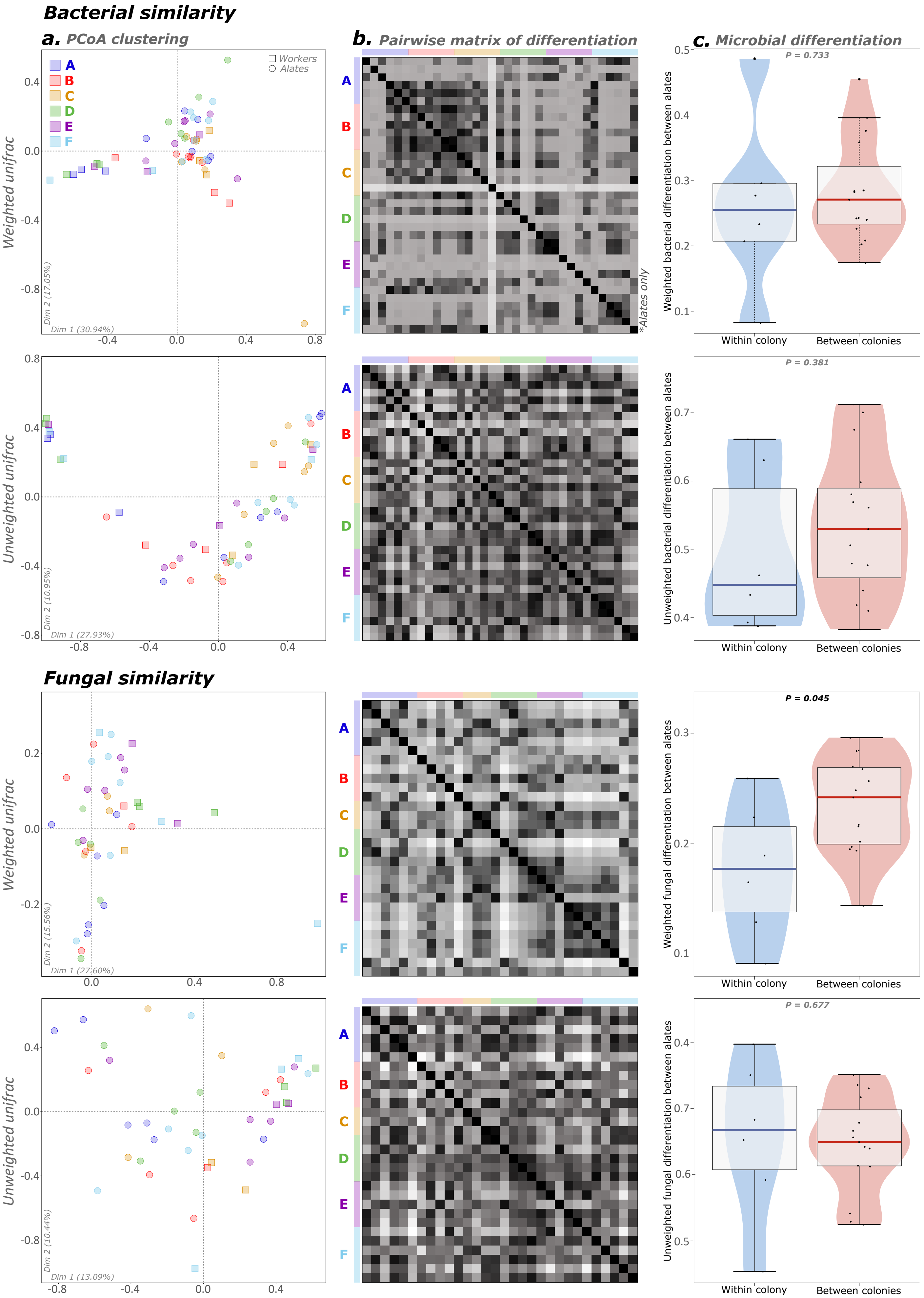

Figure S4: (a) Principal Coordinate Analyses (PCoA) of individuals based on their bacterial or fungal difference (weighted and unweighted Unifrac values). Each individual is colored according to its colony of origin, alates are indicated with circles and workers with squares. (b) Pairwise distance matrices between each pair of alates from the same or different colonies. Each pair is colored according to its microbial similarity (i.e., bacterial and fungal similarity from weighted and unweighted Unifrac) obtained from Euclidean distances between the two individuals on the two first PCs of the PCoA. Darker values indicate low microbial differentiation between a pair of individuals (i.e., close on the PCoA analysis). (c) Violin plots of bacterial and fungal differentiation (weighted and unweighted Unifrac) among individuals within and between colonies. Box plots represent median and 1st and 3rd quartile; whiskers include 95% of all observations; dots indicate individual values.

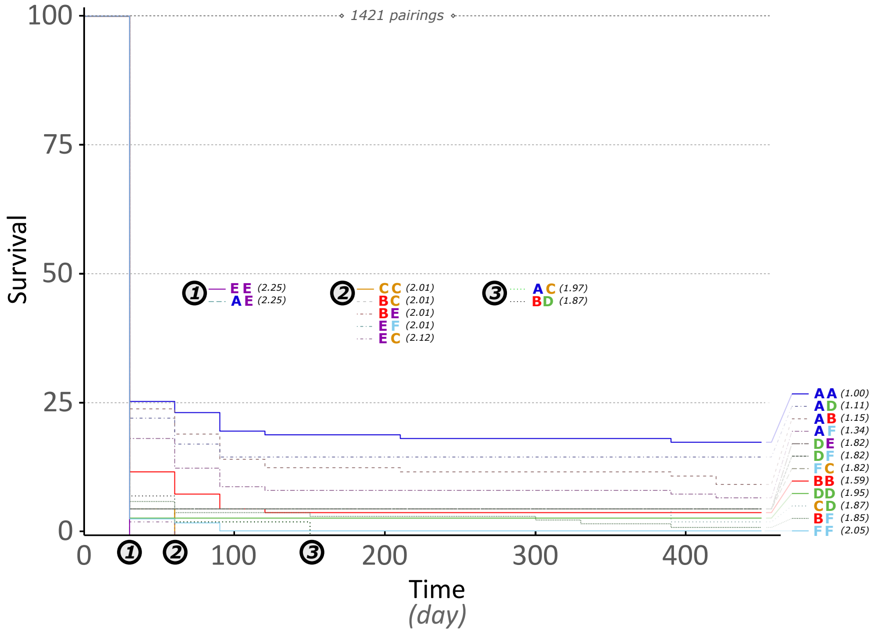

Figure S5: Survival Kaplan-Meier curves for each pairing during the overall length of the long-term survival experiment (450 days). Incipient colonies were monitored every two days. Hazard ratio for cox-proportional hazard model is reported in front of each pairing.

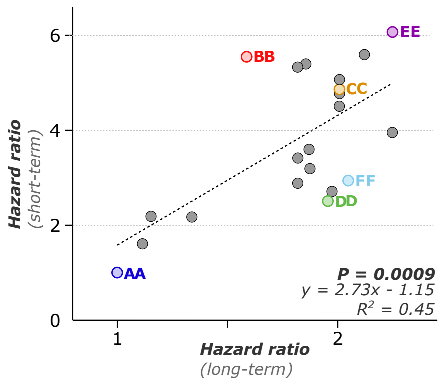

Figure S6: Correlation between hazard ratios of each pairing during the short-term experiment (14 days) and those of the long-term experiment (450 days). Hazard ratios of inbred pairings are highlighted in color.

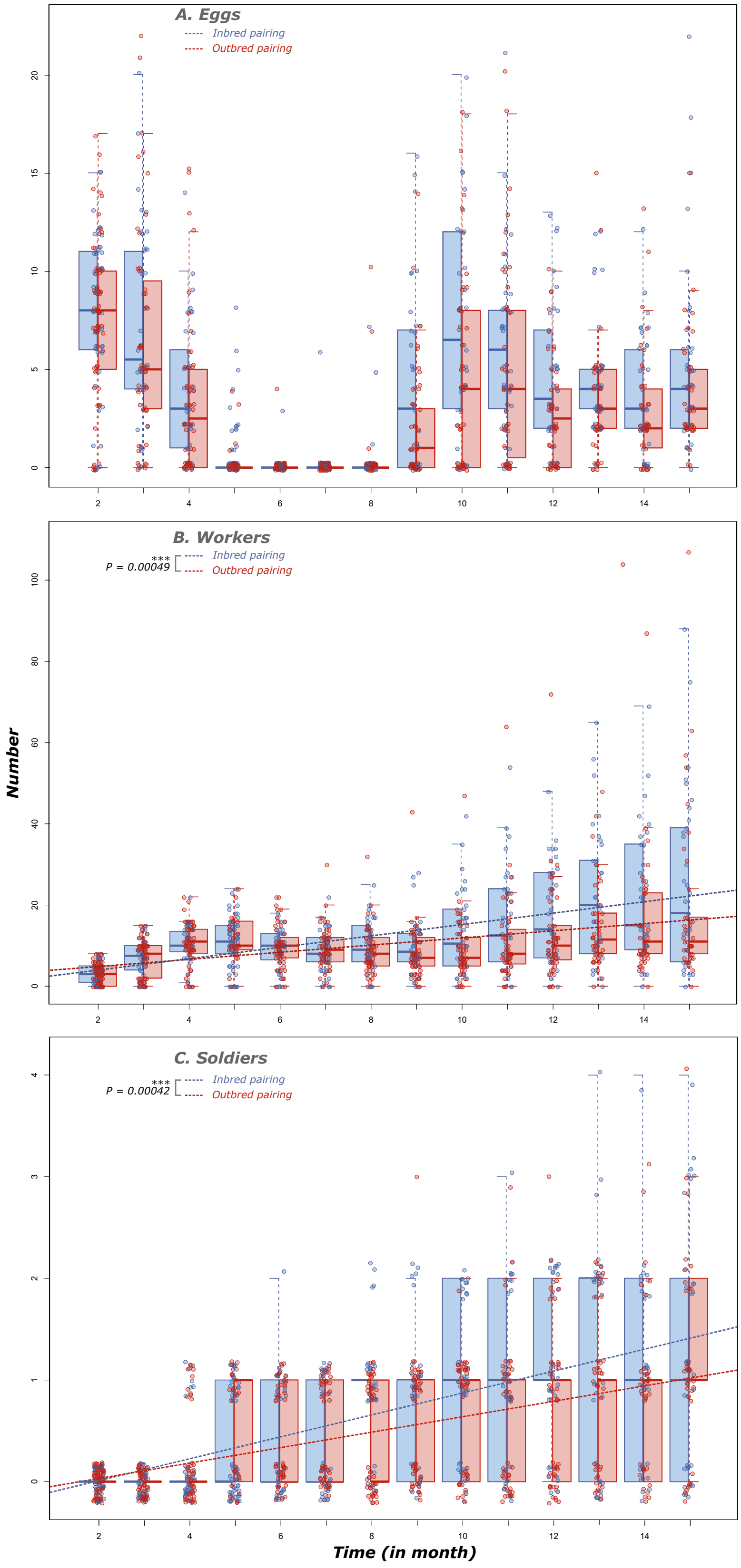

Figure S7: Number of eggs, workers and soldiers present within inbred and outbred incipient colonies each month for 450 days (15 months) after pairing.

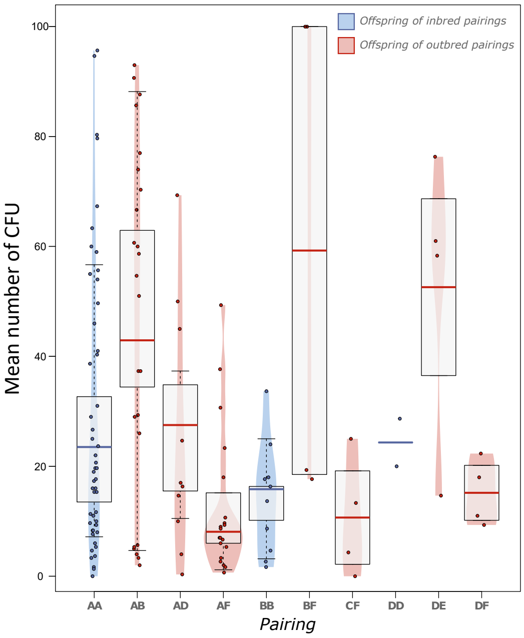

Figure S8: Microbial load (mean number of colony forming units, CFUs) for each pairing investigated.

Table S1: AIC model selection

Linear and logarithmic correlations between the susceptibility of a pairing (*i.e.,* hazard ratio) and (i) the cumulative microbial load and (ii) the maximum microbial load of the constituent

Cumulative microbial load

Linear model	Estimate	Std. Error	t-value	Pr(>|t|)	
(Intercept)	2.69991	0.40339	6.693	2.13E-06	***
cumulative microbial load	0.09957	0.02865	3.476	**0.00253**	**

F-statistic: 12.08 on 1 and 19 DF, p-value: 0.002531
Multiple R-squared: **0.3887**

Log model	Estimate	Std. Error	t-value	Pr(>|t|)	
(Intercept)	2.3905	0.3862	6.19	6.01E-06	***
log(cumulative microbial load)	0.7833	0.1751	4.474	**0.00026**	***

F-statistic: 20.02 on 1 and 19 DF, p-value: 0.0002599
Multiple R-squared: **0.5131**

	df	AIC
Linear model	3	70.47913
Log model	3	65.70196

Maximum microbial load

Linear model	Estimate	Std. Error	t-value	Pr(>|t|)	
(Intercept)	2.75453	0.46234	5.958	9.82E-06	***
Maximum microbial load	0.12683	0.04574	2.773	**0.0121**	*

F-statistic: 7.687 on 1 and 19 DF, p-value: 0.01213
Multiple R-squared: **0.288**

Log model	Estimate	Std. Error	t-value	Pr(>|t|)	
(Intercept)	2.8433	0.3425	8.301	9.63E-08	***
log(Maximum microbial load)	0.6598	0.1686	3.912	**0.000936**	***

F-statistic: 15.31 on 1 and 19 DF, p-value: 0.0009359
Multiple R-squared: **0.4462**

	df	AIC
Linear model	3	73.67978
Log model	3	68.40526
