# Supplementary material for: Short and long-term costs of inbreeding in the lifelong-partnership in a termite
Source: Commun Biol. 2022 Apr 25;5:389. doi: 10.1038/s42003-022-03317-9 (PMC9038770; doi:10.1038/s42003-022-03317-9)
Supplement: Supplementary file 3 — Reporting Summary [file 42003_2022_3317_MOESM3_ESM.pdf]

nature portfolio

Corresponding author(s): Pierre Andre Eyer

Last updated by author(s): 03/09/2022

Reporting Summary

Nature Portfolio wishes to improve the reproducibility of the work that we publish. This form provides structure for consistency and transparency in reporting. For further information on Nature Portfolio policies, see our Editorial Policies and the Editorial Policy Checklist.

Statistics

For all statistical analyses, confirm that the following items are present in the figure legend, table legend, main text, or Methods section.

n/a	Confirmed	
☐	☒	The exact sample size (n) for each experimental group/condition, given as a discrete number and unit of measurement
☐	☒	A statement on whether measurements were taken from distinct samples or whether the same sample was measured repeatedly
☐	☒	The statistical test(s) used AND whether they are one- or two-sided<br>*Only common tests should be described solely by name; describe more complex techniques in the Methods section.*
☐	☒	A description of all covariates tested
☐	☒	A description of any assumptions or corrections, such as tests of normality and adjustment for multiple comparisons
☐	☒	A full description of the statistical parameters including central tendency (e.g. means) or other basic estimates (e.g. regression coefficient) AND variation (e.g. standard deviation) or associated estimates of uncertainty (e.g. confidence intervals)
☐	☒	For null hypothesis testing, the test statistic (e.g. F, t, r) with confidence intervals, effect sizes, degrees of freedom and P value noted<br>*Give P values as exact values whenever suitable.*
☐	☒	For Bayesian analysis, information on the choice of priors and Markov chain Monte Carlo settings
☐	☒	For hierarchical and complex designs, identification of the appropriate level for tests and full reporting of outcomes
☐	☒	Estimates of effect sizes (e.g. Cohen's d, Pearson's r), indicating how they were calculated

Our web collection on statistics for biologists contains articles on many of the points above.

Software and code

Policy information about availability of computer code

Data collection	An ordinary spreadsheet software was used to collect data in this manuscript
Data analysis	The statistical analyses were performed using the open access R software

For manuscripts utilizing custom algorithms or software that are central to the research but not yet described in published literature, software must be made available to editors and reviewers. We strongly encourage code deposition in a community repository (e.g. GitHub). See the Nature Portfolio guidelines for submitting code & software for further information.

Data

Policy information about availability of data

All manuscripts must include a data availability statement. This statement should provide the following information, where applicable:

- Accession codes, unique identifiers, or web links for publicly available datasets
- A description of any restrictions on data availability
- For clinical datasets or third party data, please ensure that the statement adheres to our policy

The data reported in this study will be deposited in the Open Science Framework database upon acceptance, https://osf.io

Field-specific reporting

Please select the one below that is the best fit for your research. If you are not sure, read the appropriate sections before making your selection.

☐ Life sciences ☐ Behavioural & social sciences ☒ Ecological, evolutionary & environmental sciences

For a reference copy of the document with all sections, see nature.com/documents/nr-reporting-summary-flat.pdf

Ecological, evolutionary & environmental sciences study design

All studies must disclose on these points even when the disclosure is negative.

Study description	We prepared 40 outbred pairings for every combination of termite colonies, with an equal number of each sex per colony of origin (20 queensA x kingB and 20 queensB x kingA); resulting in 231 inbred and 600 outbred incipient colonies. To ensure robust sample sizes, we anticipated high mortality during colony foundation and established an additional 290 inbred and 300 outbred pairings. Overall, we set up 1421 incipient colonies (521 inbred and 900 outbred), all of which were established on the same day
Research sample	Six termite colonies of Reticulitermes flavipes collected in Bryan, TX, USA in March 2020 were used in this study.
Sampling strategy	We prepared 40 outbred pairings for every combination of termite colonies, with an equal number of each sex per colony of origin (20 queensA x kingB and 20 queensB x kingA); resulting in 231 inbred and 600 outbred incipient colonies. To ensure robust sample sizes, we anticipated high mortality during colony foundation and established an additional 290 inbred and 300 outbred pairings. Overall, we set up 1421 incipient colonies (521 inbred and 900 outbred), all of which were established on the same day
Data collection	Microbial loads were estimated from the number of colony forming units (CFUs) cultured from individual cuticular washes of 12 alates (6 females and 6 males) and 6 workers per colony. The survival of incipient colonies was recorded by the first author.
Timing and spatial scale	The survival of the 231 inbred and 600 outbred colonies was assessed every two days for 14 days after pairing. . The survival of the 1421 incipient colonies (521 inbred and 900 outbred) was assessed every month for 15 months.
Data exclusions	No data were excluded
Reproducibility	All attempts to repeat the experiment were succesful
Randomization	The models test the relationship between the numbers of workers and soldiers present in colonies as a function of time (fixed effect), with the type of pairing (inbred or outbred) tested as a random effect.
Blinding	Blinding was not possible as colony ID was required for the analyses of this study

Did the study involve field work? ☐ Yes ☒ No

Reporting for specific materials, systems and methods

We require information from authors about some types of materials, experimental systems and methods used in many studies. Here, indicate whether each material, system or method listed is relevant to your study. If you are not sure if a list item applies to your research, read the appropriate section before selecting a response.

Materials & experimental systems

n/a	Involved in the study	
☒	☐	Antibodies
☒	☐	Eukaryotic cell lines
☒	☐	Palaeontology and archaeology
☐	☒	Animals and other organisms
☒	☐	Human research participants
☒	☐	Clinical data
☒	☐	Dual use research of concern

Methods

n/a	Involved in the study	
☒	☐	ChIP-seq
☒	☐	Flow cytometry
☒	☐	MRI-based neuroimaging

Animals and other organisms

Policy information about studies involving animals; ARRIVE guidelines recommended for reporting animal research

Laboratory animals	The study did not involve lab animals
Wild animals	Stock termite colonies of Reticulitermes flavipes were collected in Bryan, TX, USA in March 2020. Colonies were extracted from their wooden logs and transferred into 20cm plastic boxes.

Field-collected samples	Colonies were extracted from their wooden logs and transferred into 20cm plastic boxes. The incipient colonies were in the dark in kept in high humidity chambers at room temperature.
Ethics oversight	No ethical approval or guidance was required as this study involves insects. Moreover, the termite species used in this study is consider as a pest nuisance, and is a invasive species. This species is therefore not considered of concern for biological conservation

Note that full information on the approval of the study protocol must also be provided in the manuscript.
